# Bidirectional plasticity of GABAergic tonic inhibition in hippocampal somatostatin- and parvalbumin-containing interneurons

**DOI:** 10.3389/fncel.2023.1193383

**Published:** 2023-06-28

**Authors:** Marcin Wyroślak, Grzegorz Dobrzański, Jerzy W. Mozrzymas

**Affiliations:** ^1^Department of Biophysics and Neuroscience, Wroclaw Medical University, Wrocław, Poland; ^2^Nencki Institute of Experimental Biology, Warsaw, Poland

**Keywords:** GABA, tonic inhibition, hippocampus, interneurons, plasticity, extrasynaptic receptors

## Abstract

GABA_A_ receptors present in extrasynaptic areas mediate tonic inhibition in hippocampal neurons regulating the performance of neural networks. In this study, we investigated the effect of NMDA-induced plasticity on tonic inhibition in somatostatin- and parvalbumin-containing interneurons. Using pharmacological methods and transgenic mice (SST-Cre/PV-Cre x Ai14), we induced the plasticity of GABAergic transmission in somatostatin- and parvalbumin-containing interneurons by a brief (3 min) application of NMDA. In the whole-cell patch-clamp configuration, we measured tonic currents enhanced by specific agonists (etomidate or gaboxadol). Furthermore, in both the control and NMDA-treated groups, we examined to what extent these changes depend on the regulation of distinct subtypes of GABA_A_ receptors. Tonic conductance in the somatostatin-containing (SST+) interneurons is enhanced after NMDA application, and the observed effect is associated with an increased content of α5-containing GABA_A_Rs. Both fast-spiking and non–fast-spiking parvalbumin-positive (PV+) cells showed a reduction of tonic inhibition after plasticity induction. This effect was accompanied in both PV+ interneuron types by a strongly reduced proportion of δ-subunit-containing GABA_A_Rs and a relatively small increase in currents mediated by α5-containing GABA_A_Rs. Both somatostatin- and parvalbumin-containing interneurons show cell type-dependent and opposite sign plasticity of tonic inhibition. The underlying mechanisms depend on the cell-specific balance of plastic changes in the contents of α5 and δ subunit-containing GABA_A_Rs.

## Highlights

- NMDA induces plastic changes of tonic inhibition in hippocampal interneurons
- Tonic current plasticities in SST+ and PV+ interneurons show opposite directions
- α5- and δ-GABA_A_R contents were altered upon plasticity induction

## 1. Introduction

GABAergic inhibition consists of two major components: tonic and phasic drives. While phasic signaling has been mediated by GABA_A_ receptors (GABA_A_Rs) located at postsynaptic densities, tonic inhibition (TI) relies on high-affinity receptors present in the extrasynaptic regions (Farrant and Nusser, 2005). Ambient GABA can activate extrasynaptic GABA_A_Rs and cause their persistent conductance at low, often submicromolar concentrations (Lerma et al., 1986; Kaneda et al., 1995; Brickley et al., 1996). Receptors that mediate tonic inhibition typically include α(4-6), β, δ (for α4/6), or γ (for α5) subunits, but it needs to be considered that distinct types of neurons in various brain regions may express GABA_A_Rs with different subunit stoichiometry (Caraiscos et al., 2004; Serwanski et al., 2006; Glykys et al., 2008; Brickley and Mody, 2012; Field et al., 2021). Importantly, a growing body of evidence has demonstrated that TI plays an important role in learning and memory depending on the hippocampus. Moreover, the use of knockout Gabra5(–/–) mice showed that the GABA_A_R-containing α5 subunit was involved in modulating the hippocampal-dependent memory (Martin et al., 2009). The δ subunit was present in the hippocampus, although its expression depends on the neuronal type (Sun et al., 2004; Mangan et al., 2005; Glykys et al., 2008), and it has been shown that δ subunit-containing GABA_A_Rs are essential in learning and memory formation (Whissell et al., 2013a; Cushman et al., 2014).

For decades, research on excitatory glutamatergic synaptic plasticity was the primary focus; but in the past years, there has been a significant increase in interest in GABAergic inhibitory plasticity. Examples of NMDAR-dependent GABAergic plasticity have been described in the cerebral cortex (Chiu et al., 2018) and in the hippocampus (Marsden et al., 2007; Wiera et al., 2021, 2022). Moreover, several studies have shown that α5 subunit-containing GABA_A_Rs were involved in the regulation of glutamatergic plasticity (Cheng et al., 2006; Ballard et al., 2009; Martin et al., 2010; Davenport et al., 2021). One of our recent studies indicates the involvement of tonically active α5-containing GABA_A_Rs in the NMDA-induced plastic changes of the tonic inhibition in the pyramidal cells (Wyroślak et al., 2021). However, the abovementioned evidence was based on recordings primarily from pyramidal neurons. It is thus appealing to explore the plasticity of the tonic drive in GABAergic interneurons, which show a large diversity in innervating patterns of the principal cells and other interneurons (Pelkey et al., 2017). We thus decided to address the issue of tonic inhibition plasticity at distinct types of interneurons, known to play a crucial role in regulating the hippocampal neuronal network. Parvalbumin-containing (PV+ INs) and somatostatin-containing (SST+ INs) interneurons were known to have a profound impact on hippocampal activity while innervating distinct parts of pyramidal neurons—PV+ INs—perisomatic, SST+ INs—distal dendrites (Figure 4 and also Pelkey et al., 2017). Recent studies have shown that parvalbumin-containing interneurons are critical in memory consolidation by the coordination of neural network dynamics (Donato et al., 2013; Ognjanovski et al., 2017; Udakis et al., 2020). Moreover, SST+ INs were found to play an important role in regulating neuronal activity, plasticity, and pathology (Leão et al., 2012; Honoré et al., 2021; Asgarihafshejani et al., 2022; Liguz-lecznar et al., 2022). We report here that brief NMDA stimulation induces in these INs the cell type-specific plastic changes in tonic inhibition with opposite signs (PV+ INs – reduction, SST+ INs – potentiation). While the plasticity in PV+ INs appears to be associated primarily with the reduction of the tonically active δ subunit-containing GABA_A_Rs in SST+ Ins, it results in an increased content of α5GABA_A_Rs in GABAergic tonic inhibition.

## 2. Materials and methods

### 2.1. Ethical approval

All animal care and experimental procedures were conducted in the animal facility of the Wroclaw Medical University in accordance with the European Community Council Directive (2010/63/UE). Before decapitation, mice were anesthetized with isoflurane. All efforts were made to minimize the number of animals used.

### 2.2. Animals

Animals were housed on a natural light/dark cycle (12/12 h) with food and water *ad libitum*. Experiments were performed on 18–25-day-old mice of either sex. Wild-type mice and homozygous knock-in mice expressing Cre recombinase (PV-Cre; JAX 017320 and SST-Cre; JAX 028864) crossed with Rosa26-tdTomato reporter mice (Ai14; JAX 007914) were used.

### 2.3. Brain slices preparation

Mice were anesthetized with isoflurane and then euthanized by decapitation. Brains were placed in a cold artificial cerebrospinal fluid (aCSF) containing 119 mM NaCl, 2.5 mM KCl, 1 mM NaH_2_PO_4_, 26.3 mM NaHCO_3_, 1.3 mM MgSO_4_, 2.5 mM CaCl_2_, and 11 mM glucose, and a pH of 7.4 bubbled with carbogen (95% O_2_ + 5% CO_2_). Brains were cut with a vibratome (Leica VT1200S, Germany) into 350-μm-thick transverse slices, in which the hippocampus was easily visible. After sectioning, slices were transferred to a recovery chamber containing aCSF for at least 1 h before electrophysiological experiments.

### 2.4. Drugs

The following drugs were purchased from Tocris Bioscience (UK) and were used during the experiments: 6,7-dinitroquinoxaline-2,3-dione (DNQX; selective blocker of non-NMDA glutamate receptors), tetrodotoxin (TTX; sodium channel selective blocker), gaboxadol (THIP; superagonist for δ-containing extrasynaptic GABA_A_Rs), etomidate (enhancer of β2/3-containing GABA_A_Rs), L-655,708 (selective inverse agonist for α5-containing GABA_A_Rs), picrotoxin (PTX; non-specific GABA_A_Rs antagonist), and N-methyl-D-aspartic acid (NMDA; NMDARs selective agonist). The stock solutions of etomidate, L-655,708, and picrotoxin were dissolved in dimethyl sulfoxide (DMSO, Sigma) and then added into the ACSF during experiments [not exceeding the concentrations of DMSO > 0.1% v/v (see Lebida and Mozrzymas, 2017)].

### 2.5. Electrophysiological recordings and data analysis

Prior to measurements, slices were transferred to a recording chamber perfused with oxygenated aCSF at a flow rate of 2.0–3.0 ml/min at room temperature. Both, parvalbumin- and somatostatin-containing interneurons were identified based on tdTomato expression visualized by fluorescence microscopy equipped with Lambda DG-4, an illumination system designed for a rapid change in wavelength (Sutter Instrument).

PV+ interneurons were searched in the stratum pyramidale as cells with large, pyramidally shaped or bitufted dendritic trees. Interneurons were classified as fast-spiking INs if high firing frequency (67.88 ± 3.26 Hz), narrow spike half widths (0.62 ± 0.03 ms), and short membrane time constants (9.62 ± 1.35 ms) are displayed. Non–fast-spiking interneurons were characterized by lower firing frequencies (21.54 ± 1.81 Hz), wider spike half widths (1.32 ± 0.03 ms), and longer membrane time constants (23.10 ± 1.24 ms) (Pelkey et al., 2017). The membrane and firing properties for all considered interneurons are summarized in Table 1. A sag ratio was calculated from a voltage response to −200 pA using [(1 – Δ*V*_*ss*_/Δ*V*_max_) × 100%] as described by Song et al. (2015). The threshold for action potential generation was defined as the value of the cell membrane potential at which dV/dt = 20 mV/ms. The afterhyperpolarization (AHP) amplitude was determined as the difference between the action potential threshold and the least positive membrane potential immediately after the first action potential. SST+ interneurons were located in CA1 stratum oriens, parallel to the stratum pyramidale, with an apparently expanded dendritic tree. Patch-clamp recordings of tonic currents were performed in the whole-cell configuration using borosilicate patch pipettes filled with an intracellular solution containing: 10 mM potassium gluconate, 125 mM KCl, 1 mM EGTA, 10 mM HEPES, 4 mM MgATP, 5 mM sucrose, pH 7.25, 295 mOsm (Marsden et al., 2007) which had a resistance of 2.5–4.5 mOhm (when filled with internal saline). Recordings were digitized at 20 kHz and filtered at 6 kHz using a Multi-Clamp 700B amplifier and Axon Digidata 1550 (Molecular Devices).

**Table 1:** Summary of intrinsic membrane and firing properties of SST+, fast-spiking, and non–fast-spiking interneurons.

**Property**	**SST + INs**	**PV + fast spiking INs**	**PV+ non–fast-spiking INs**
	**(*****n*** = **37)**	**(*****n*** = **36)**	**(*****n*** = **37)**
RMP (mV)	−55.72 ± 1.67	−54.42 ± 1.01	−59.19 ± 2.28
Membrane time constant (ms)	45.52 ± 2.17	9.62 ± 1.35	23.10 ± 1.24
Firing frequency (Hz)	40.78 ± 2.31	67.88 ± 3.26	21.54 ± 1.81
AP amplitude (mV)	71.45 ± 1.29	57.92 ± 2.37	83.01 ± 2.22
Spike half-width (ms)	0.87 ± 0.02	0.62 ± 0.03	1.32 ± 0.03
Sag ratio	16.29 ± 2.34	6.12 ± 0.58	13.38 ± 0.87
AHP amplitude (mV)	−26.05 ± 0.85	−23.82 ± 1.02	−9.81 ± 0.77
AP threshold (mV)	−42.02 ± 0.75	−39.32 ± 1.36	−42.15 ± 1.15
Membrane capacitance (pF)	84.76 ± 4.46	80.93 ± 5.88	118.82 ± 7.89
Firing pattern			
	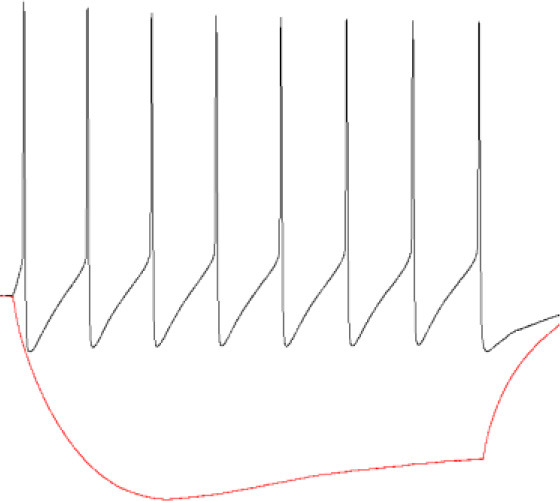	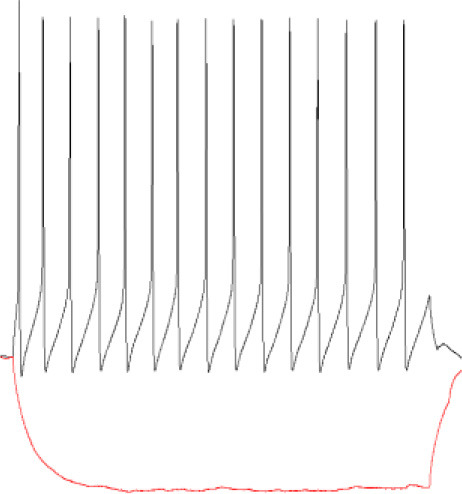	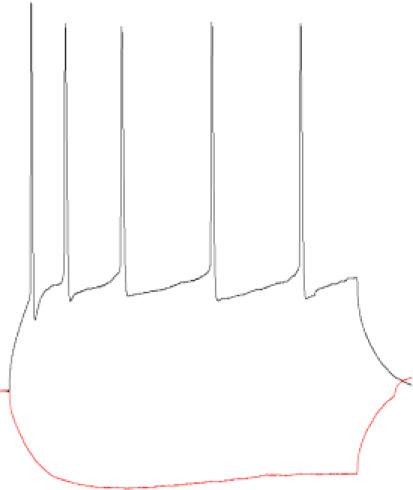

Data are represented as mean ± SEM.

After firing pattern recordings, TTX (1 μM) and DNQX (20 μM) were used to block action potentials dependent on voltage-gated sodium channels and AMPA-type glutamate receptors. These compounds remained in the measuring chamber until the end of the experiment. Furthermore, we induced plasticity in studied groups of cells by treating the slice for 3 min with 20 μM NMDA. After a stable 20 min, measurement after NMDA washout, gaboxadol (1 μM), or etomidate (5 μM) was used to enhance tonic current in PV+ and SST+ interneurons. In the final part of the experiments, PTX (100 μM) was administered to silence GABAergic transmission.

In this study, miniature inhibitory postsynaptic currents (mIPSCs) were excluded from the analysis to precisely determine the magnitude of tonic currents (as recommended in Bright and Smart, 2013). The tonic current density was determined from the current shift following PTX application, normalized to the whole-cell membrane capacitance (Cm). The capacitance of the cell membrane was determined as a ratio of the membrane time constant and the input resistance. The membrane time constant was estimated from the exponential fit to the time course of the membrane voltage (in current-clamp mode) in response to a small −25 pA hyperpolarizing current (Urban-Ciecko et al., 2010; Wyroślak et al., 2021). Access and input resistances were monitored during the recordings. Cells were discarded from further analysis if the monitored resistances changed by >20%.

To evaluate the contribution of α5GABA_A_Rs in the total tonic density values, after etomidate/gaboxadol administration, we additionally used L-655,708 before the application of PTX. The proportion of current mediated by α5GABA_A_Rs was calculated for each cell as ΔIL-655,708/(ΔIL-655,708 + ΔIPTX), where ΔIL-655,708 and ΔIPTX are current reductions following the administration of L-655,708 and PTX, respectively. To ensure the specificity of action on α5-GABA_A_Rs, a low concentration of L-655,708 of 20 nM was used as its higher concentrations might affect the activity of other synaptic or extrasynaptic GABA_A_Rs (Atack et al., 2006; Vargas-Caballero et al., 2010).

### 2.6. Experimental design and analysis

Studies were designed to generate equal-size groups, and the brain slices were randomized for treatment. Brain slices were isolated from both male and female subjects in each considered group. The results were combined because there were no differences or trends between the sexes. The group size (*n*) for each group was collected to obtain a relevant power of the statistical analysis (β > 0.8). Group sizes indicate the number of experimentally determined values (each value refers to one cell in a separate brain slice). SigmaPlot (Systat software) was used to perform data analysis. Data on the plots are presented as mean ± SEM. The data was checked for normal distributions (Kolmogorov–Smirnov test) and equal variances (Levene median test). Comparisons were performed using unpaired or paired Student's *t*-test. Differences were considered statistically significant when the value of *p* < 0.05 was obtained. Randomization or blinding of the operator or data analysis was not undertaken due to the nature of the experiments.

## 3. Results

In our previous study (Wyroślak et al., 2021), we found that brief (3 min) NMDA treatment induced plasticity of tonic GABAergic currents in the pyramidal neurons. We thus applied an analogous protocol to investigate the plasticity of tonic conductance in three subtypes of interneurons: somatostatin-positive and two types of parvalbumin-containing cells: fast-spiking and non-fast spiking. Because of relatively intense superfusion of slices with aCSF, which is expected to reduce the ambient GABA (Glykys and Mody, 2007; Mody et al., 2007), we have used protocols to enhance tonic currents with etomidate or gaboxadol as also practiced in our previous (Wyroślak et al., 2021) and other studies (Martin et al., 2009; Rodgers et al., 2015; Zarnowska et al., 2015).

### 3.1. Transient activation of NMDARs enhances tonic current in SST+ interneurons

**Figure 1:**
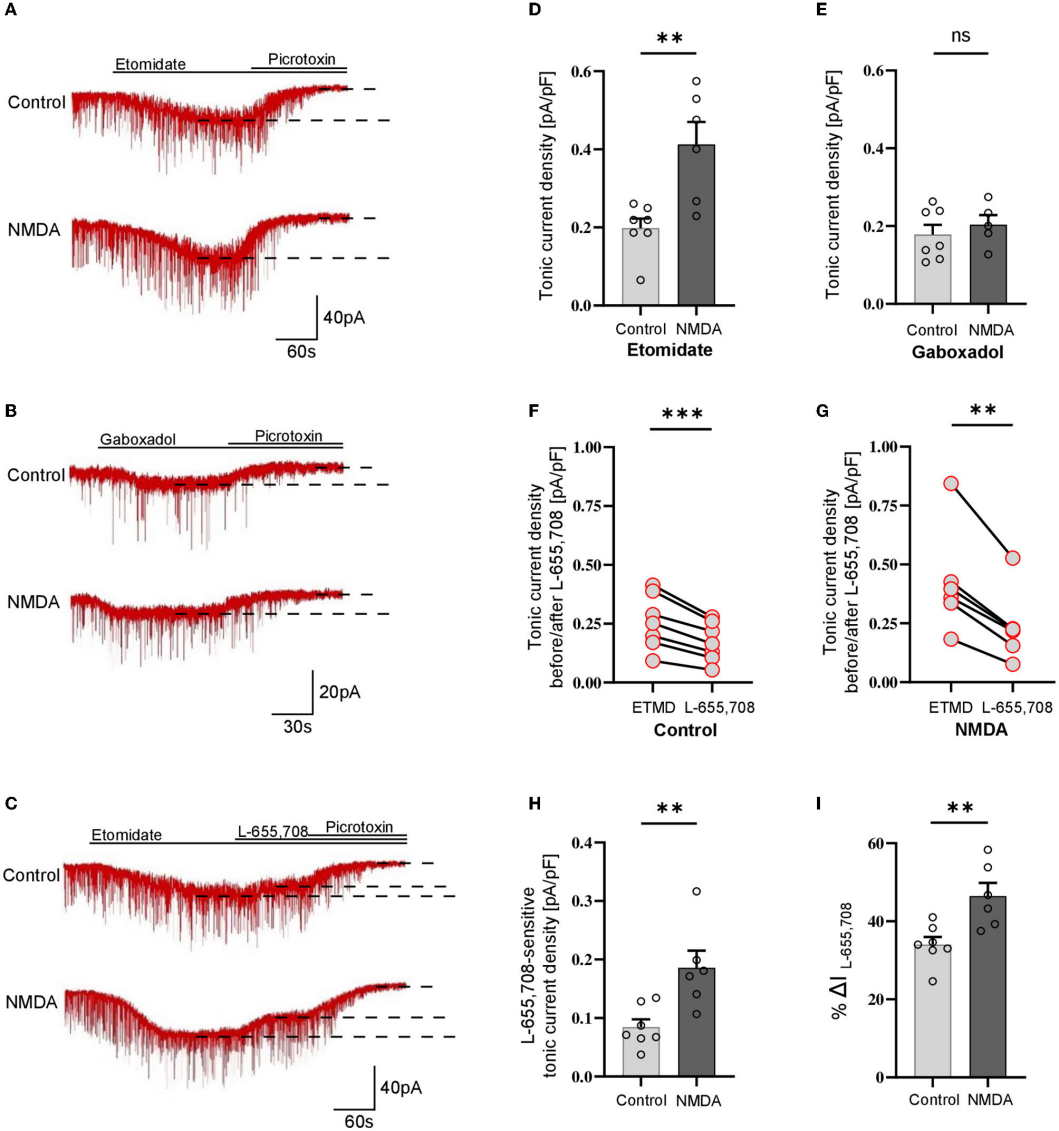
NMDA-dependent plasticity induction enhances GABAergic tonic current in CA1 somatostatin-positive interneurons. **(A–C)** Representative tonic current traces in the control **(top)** and 3-min NMDA-treated groups **(bottom)**. TTX and DNQX were used during the measurements. Insets above current traces indicate applications of different pharmacological compounds. **(D, E)** Statistics for tonic current density enhanced with etomidate **(D)** or gaboxadol **(E)** in control conditions (gray) and after NMDA treatment (black). **(F, G)** The effect of L-655,708 administration on tonic current density enhanced by etomidate in the control **(F)** or NMDA-treated **(G)** groups. **(H)** Statistical comparison for the absolute values of L-655,708-sensitive current component in the control (gray) and NMDA-treated (black) groups. **(I)** Statistics for the mean percentage of L-655,708-sensitive current in total tonic current density [%ΔI (L-655,708)] in control (gray) and NMDA-treated group (black). Analysis was conducted with the unpaired and paired *t*-test. **p < 0.01, ***p < 0.001, ns, non-significant. Data on the plots are presented as mean ± SEM and circles represent values collected in recordings from separate cells.

As explained above, tonic currents were enhanced either with etomidate (5 μM) or gaboxadol (1 μM). The tonic current density was determined from the subtraction of the steady-state current measured upon PTX (100 μM) treatment from that evoked by etomidate (or gaboxadol) (Figures 1A, B) and by normalizing these values to the whole-cell membrane capacitance (Figures 1D–I). Interestingly, we found that the average etomidate-enhanced tonic current density was significantly increased by NMDA treatment (Control: 0.198 ± 0.07 pA/pF, *n* = 7; NMDA: 0.413 ± 0.14, *n* = 6; *p* = 0.004; Figures 1A, D). We used additionally gaboxadol instead of etomidate to check whether there is a component of plastic changes associated with GABA_A_Rs with δ subunit but we did not observe any significant difference between the control and NMDA-treated groups (Control: 0.178 ± 0.07, *n* = 7; NMDA: 0.204 ± 0.06, *n* = 5; *p* = 0.498; Figures 1B, E), indicating that δGABA_A_Rs are not involved in the tonic current plasticity in these neurons. Next, we sought to determine the contribution of α5GABA_A_Rs in observed plastic changes. To this end, we first elicited the tonic current with etomidate, and when it reached the steady state, a specific blocker of α5 subunit-containing GABA_A_Rs (L-655,708) was applied (Figure 1C). Administration of this compound resulted in a clear reduction of tonic currents both in the control (ETMD: 0.258 ± 0.04 pA/pF; L-655,708: 0.173 ± 0.03 pA/pF; *n* = 7; *p* < 0.001; Figure 1F) and NMDA-treatment groups (EMTD: 0.424 ± 0.09 pA/pF; L-655,708: 0.238 ± 0.06 pA/pF; = 6; *p* = 0.001; Figure 1G), but a significantly larger reduction of tonic current was observed in the NMDA group (Control: 0.084 ± 0.01 pA/pF, *n* = 7; NMDA: 0.186 ± 0.03 pA/pF, *n* = 6; *p* < 0.01; Figure 1H). Then, the fraction of current sensitive to L-655,708 (%ΔIL-655,708) was calculated as described in Methods, and as shown in Figure 1I, plasticity induction with NMDA was associated with significantly increased contribution of current mediated by the α5GABA_A_Rs [Control: 34.025 ± 1.95 %ΔI (L-655,708), *n* = 7; NMDA: 46.486 ± 3.35 %ΔI (L-655,708), *n* = 6; *p* < 0.05; Figure 1I]. We thus provide the first evidence that a brief NMDA treatment induced tonic current plasticity in SST+ interneurons and that these changes are related to increased content of α5GABA_A_Rs in the mediation of extrasynaptic GABAergic currents.

### 3.2. NMDA treatment reduces GABAergic tonic currents in parvalbumin-containing fast-spiking and non–fast-spiking interneurons

**Figure 2:**
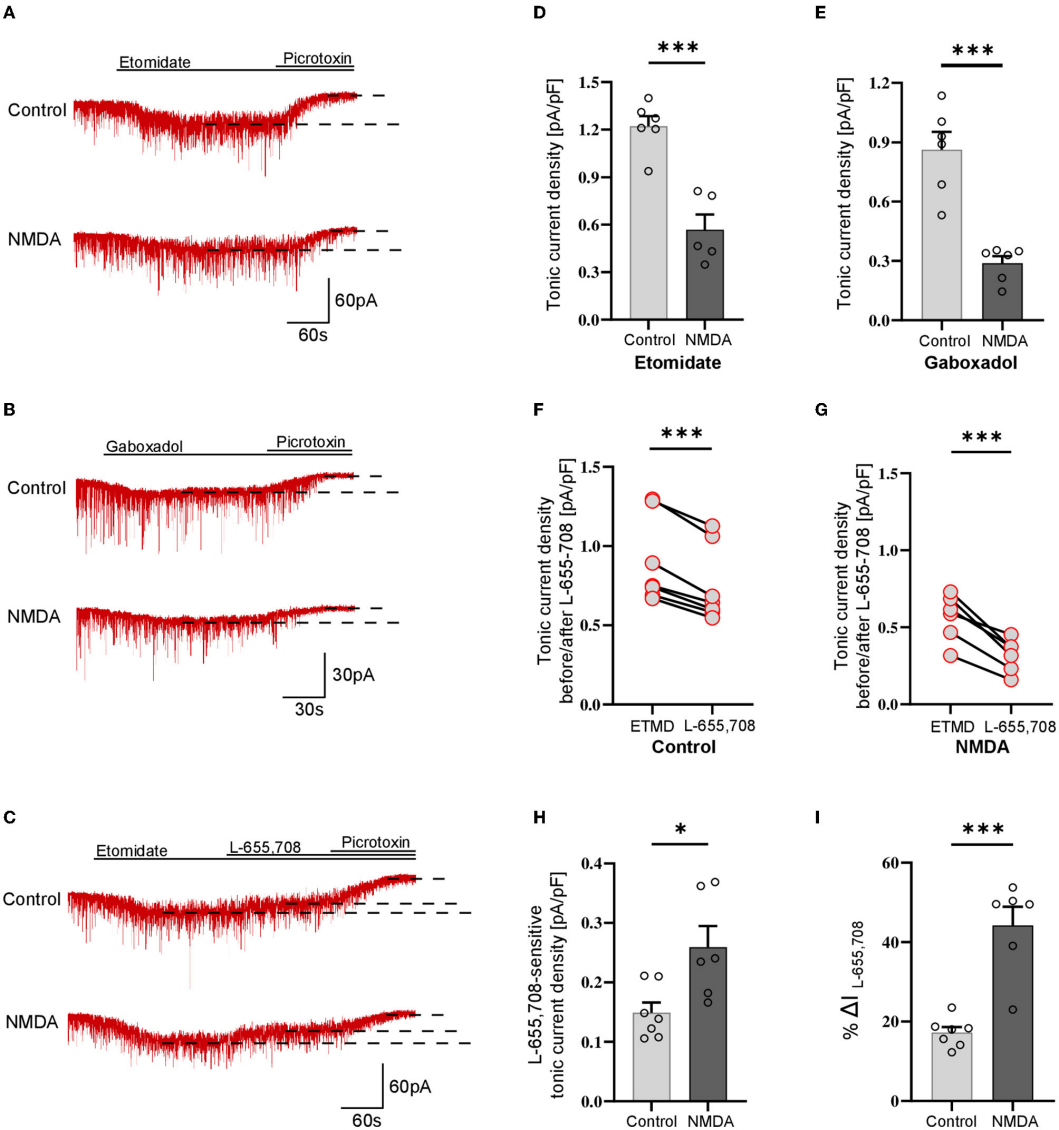
NMDA treatment reduces GABAergic tonic conductance in parvalbumin-containing fast-spiking interneurons. **(A–C)** Representative tonic current traces in the control **(top)** and 3-min NMDA-treated **(bottom)** groups. TTX and DNQX were used during the measurements. Insets above current traces indicate applications of different pharmacological compounds. **(D, E)** Statistics for tonic current density enhanced with etomidate **(D)** or gaboxadol **(E)** in control conditions (gray) and after NMDA treatment (black). Note that, in contrast to SST+ interneurons (Figure 1), NMDA treatment reduces tonic currents. **(F, G)** The effect of L-655,708 administration on tonic current density enhanced by etomidate in the control **(F)** or NMDA-treated **(G)** groups. **(H)** Statistics for the L-655,708-sensitive current component in the control (gray) and NMDA-treated (black) groups. **(I)** Statistics for the mean percentage of L-655,708-sensitive current [%ΔI (L-655,708)] in control (gray) and NMDA-treated (black) groups. Analysis was conducted with the unpaired and paired *t*-test. *p < 0.05, ***p < 0.001, ns, non-significant. Data on the plots are presented as mean ± SEM and circles represent values collected in recordings from separate cells.

**Figure 3:**
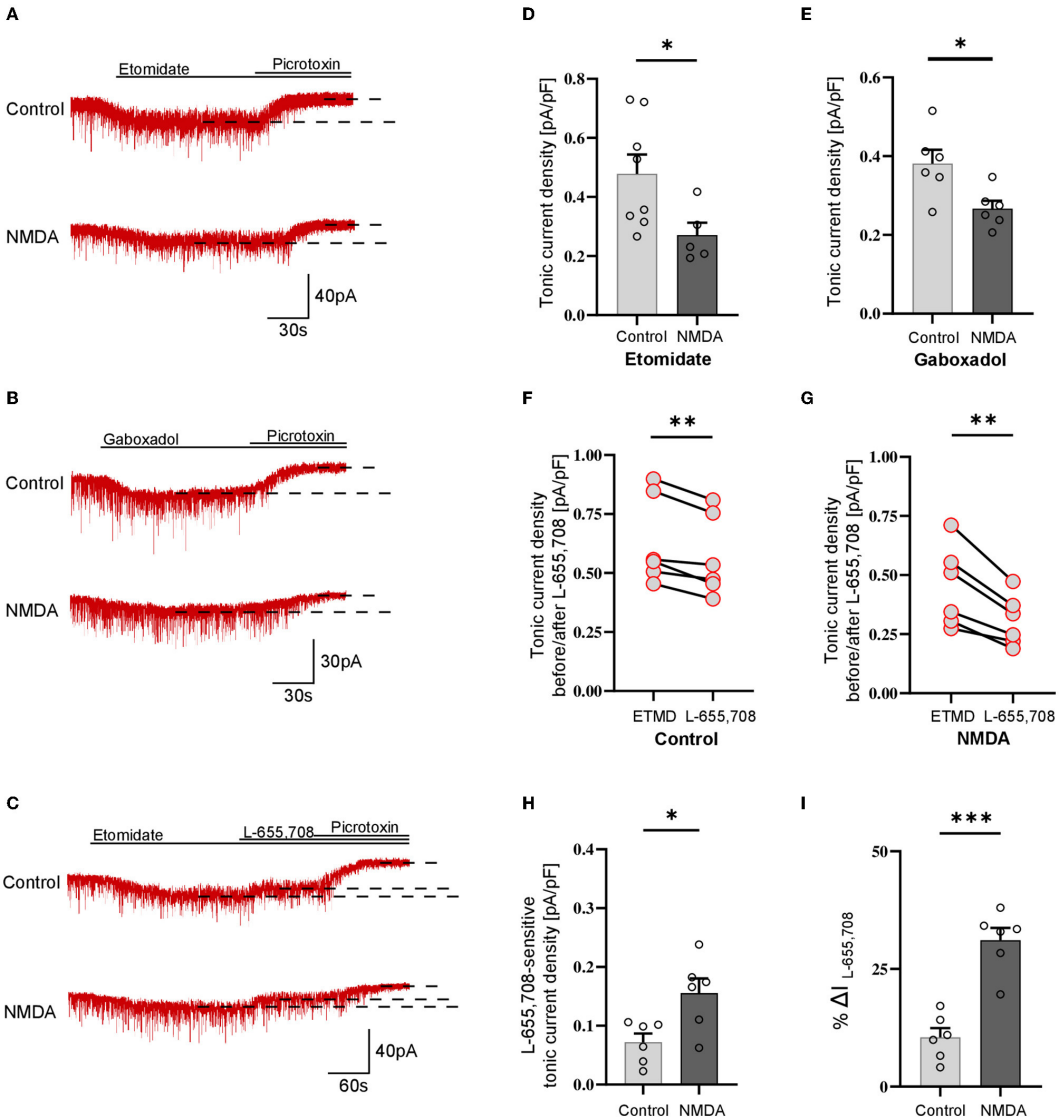
Activation of NMDARs decreases tonic current in PV+ non–fast-spiking interneurons. **(A–C)** Representative tonic current traces in control **(top)** and 3 min NMDA-treated group **(bottom)**. TTX and DNQX were used during the measurements. Insets above current traces indicate applications of different pharmacological compounds. **(D, E)** Statistics for tonic current density enhanced with etomidate **(D)** or gaboxadol **(E)** in control conditions (gray) and after NMDA treatment (black). Note that these results are similar to those obtained for the fast-spiking PV+ interneurons (Figure 2) and opposite to those reported for SST+ interneurons (Figure 1). **(F, G)** The effect of L-655,708 administration on tonic current density enhanced by etomidate in the control **(F)** or NMDA-treated **(G)** groups. **(H)** Statistics for the L-655,708-sensitive current component in the control (gray) and NMDA-treated groups (black). **(I)** Statistics for the mean percentage of L-655,708-sensitive current [%ΔI (L-655,708)] in the control (gray) and NMDA-treated groups (black). Analysis was conducted with the unpaired and paired *t*-test. *p < 0.05, **p < 0.01, ***p < 0.001. Data on the plots are presented as mean ± SEM and circles represent values collected in recordings from separate cells.

Analogous protocol as in the above-described experiments on SST-positive neurons was applied to check for the plasticity of tonic currents in parvalbumin-containing interneurons. As described in Methods, PV+ interneurons were divided into two groups: fast-spiking (Figure 2) and non-fast spiking (Figure 3). Contrary to SST+ interneurons, in the case of PV+ fast-spiking neurons, NMDA treatment resulted in a significant reduction of tonic current density (Control: 1.222 ± 0.16 pA/pF, *n* = 6; NMDA: 0.568 ± 0.21 pA/pF, *n* = 5; *p* = 0.001; Figures 2A, D). Interestingly, a decrease in tonic current following NMDA application was also observed when gaboxadol was used (Control: 0.862 ± 0.22, *n* = 6; NMDA: 0.275 ± 0.04, *n* = 6; *p* < 0.001; Figures 2B, E), indicating that the observed plasticity was associated with a reduced number of δ subunit-containing GABA_A_Rs in the plasma membrane. In addition, we checked the content of α5GABA_A_Rs in tonic current density measured from PV+ fast-spiking interneurons. As shown in Figure 2C, application of L-655,708 reduced the tonic current both in control conditions (ETMD: 0.875 ± 0.09 pA/pF; L-655,708: 0.726 ± 0.08 pA/pF; *n* = 7; *p* < 0.001; Figure 2F) and after NMDA treatment (ETMD: 0.598 ± 0.06 pA/pF; L-655,708: 0.339 ± 0.05 pA/pF; *n* = 6; *p* < 0.001; Figure 2G). Interestingly, a significantly larger extent of tonic current reduction was observed in the NMDA group (Control: 0.149 ± 0.02 pA/pF, *n* = 7; NMDA: 0.259 ± 0.04 pA/pF, *n* = 6; *p* = 0.014; Figure 2H), indicating that plasticity induction increases the pool of α5GABA_A_Rs in this type of PV+ interneurons. Moreover, as shown in Figure 2I, brief NMDA treatment significantly augmented the mean percentage of L-655,708-sensitive component measured as %ΔI (L-655,708) (Control: 17.229 ± 1.39, *n* = 7; NMDA: 44.223 ± 4.70, *n* = 6; *p* < 0.001; Figure 2I), and the increase in %ΔI (L-655,708) appeared to be larger than that for the absolute value of L-655,708-sensitive current (compare Figures 2H, I). Thus, while plasticity induction with NMDA in PV+ fast-spiking interneurons resulted in an overall reduction of tonic current (enhanced by etomidate), its component mediated by δ subunit-containing receptors strongly decreased but the intensity of current attributed to α5GABA_A_Rs increased.

Analogous analysis of tonic current plasticity has been performed for PV+ cells characterized as non–fast-spiking interneurons. Tonic currents enhanced with etomidate were significantly reduced by NMDA treatment (Control: 0.479 ± 0.19 pA/pF, *n* = 8; NMDA: 0.271 ± 0.09 pA/pF, *n* = 5; *p* = 0.041; Figures 3A, D), and a similar effect was observed for currents mediated by δGABA_A_Rs activated by gaboxadol (Control: 0.381 ± 0.08 pA/pF, *n* = 6; NMDA: 0.267 ± 0.05 pA/pF, *n* = 6; *p* = 0.017; Figures 3B, E). Application of L-655,708 reduced the tonic current both in the control (EMTD: 0.692 ± 0.22 pA/pF; L-655,708: 0.620 ± 0.20 pA/pF; *n* = 6; *p* = 0.004; Figure 3F) and NMDA-treated groups (EMTD: 0.486 ± 0.06 pA/pF; L-655,708: 0.331 ± 0.03 pA/pF; *n* = 6; *p* = 0.002; Figure 3G), and the extent of reduction was larger in the NMDA group (Control: 0.072 ± 0.01 pA/pF, *n* = 6; NMDA: 0.156 ± 0.03 pA/pF, *n* = 6; *p* = 0.016; Figure 3H), indicating that plasticity induction upregulated α5GABA_A_Rs. In PV+ non–fast-spiking interneurons, NMDA treatment resulted in an increased proportion of α5GABA_A_Rs [%ΔI(L-655,708)] in the tonic currents (Control: 10.483 ± 1.95, *n* = 6; NMDA: 31.105 ± 2.62, *n* = 6; *p* < 0.001; Figure 3I), and similar to what observed for fast-spiking PV+ interneurons, the increase in %ΔI (L-655,708) appeared to be larger than that for the absolute value of L-655,708-sensitive current (compare Figures 3H, I).

Taken together, we found that a brief NMDA treatment reduced tonic currents in both fast-spiking and non–fast-spiking PV-containing interneurons, and these changes were associated with the reduction of δGABA_A_Rs content and increased proportion of α5GABA_A_Rs in tonic current conductance.

## 4. Discussion

The most important finding of the present study is that tonic inhibition shows heterosynaptic, NMDAR-dependent, cell-specific plasticity in SST+ and PV+ interneurons. Importantly, these interneurons innervate distinct parts of pyramidal neurons exerting their unique regulatory roles in the principal neurons (Ognjanovski et al., 2017; Pelkey et al., 2017; Antonoudiou et al., 2020; Udakis et al., 2020). Most interestingly, the plastic phenomenon in these interneurons occurs in opposite directions: in SST+–enhancement and in the two types of PV+ cells—reduction of tonic conductance. Intriguingly, while fast-spiking and non–fast-spiking interneurons showed dramatically different patterns of excitability, their NMDAR-dependent plasticity of tonic conductance described here did not show any clear difference. Moreover, the regulation of tonic currents may depend on the contributions of δ- and α5 subunit-containing receptors. In the present study, NMDA-induced changes in the two types of interneurons were associated with different regulations of proportions of these receptor subtypes. Whereas, in the case of SST+ neurons, NMDA treatment did not affect the component of δ subunit-containing (gaboxadol-activated) but increased the α5GABA_A_Rs content. In PV+ neurons, gaboxadol-sensitive current strongly decreased and the fraction of α5GABA_A_Rs increased, which may be related to α5GABA_A_Rs exocytosis. Since in the PV+ interneurons, the overall etomidate-enhanced tonic current was reduced upon NMDA treatment, our data suggest that the downregulation of tonically active δ subunit-containing GABA_A_Rs was predominant. However, we cannot exclude that observed tonic current could also include the contribution of other subtypes of GABA_A_Rs. It is also worth mentioning that, as described in Methods, we used a relatively low concentration of L-655,708 (20 nM) to avoid non-specific blockade of other GABA_A_R subtypes. It is thus likely that the real proportion of α5GABA_A_Rs in our model was higher than that indicated by the current drop upon L-605,708 administration. It is worth also noting that, due to a strong decrease in the overall tonic current upon NMDA treatment of PV+ cells (Figure 3D), a relatively small increase in α5GABA_A_Rs content (Figure 3H) gave rise to a highly enhanced proportional contribution of these receptors [measured as %ΔI (L-655,708), Figure 3I], making the tonic inhibition substantially more dependent on α5GABA_A_Rs than in control conditions.

In our previous report, we have confirmed that the tonic current component mediated by δ subunit-containing GABA_A_Rs in pyramidal neurons is small and that it is not undergoing plastic changes induced by NMDA application (Wyroślak et al., 2021). It is thus particularly interesting that, as we show in the present report, in the case of PV+ interneurons, the tonic conductance plasticity is strongly dependent on δGABA_A_Rs. The relatively small tonic current recorded in pyramidal cells and enhanced by the administration of 1μM gaboxadol (~0.17 pA/pF, Wyroślak et al., 2021) contrasts with the several times larger tonic currents in PV+ fast-spiking interneurons measured under the same conditions (~0.86 pA/pF, Figure 2E). Moreover, PV+ non-fast spiking also exhibited greater δGABA_A_R-dependent tonic conduction (~0.38 pA/pF, Figure 3E). It is particularly interesting that δGABA_A_R-dependent tonic current in SST+ interneurons was comparable to the magnitude of tonic currents in pyramidal cells examined in the recent study (~0.18 pA/pF, Figure 1E). These findings appear to be mostly consistent with the results obtained by Lee and Maguire (2013), who reported that extrasynaptic, δ-subunit-containing GABA_A_Rs play a major role in mediating tonic GABAergic inhibition in hippocampal interneurons. Moreover, Lee and Maguire reported that the disinhibition of interneurons related to the inactivation of tonic currents resulted in substantial alterations in the neuronal excitability of pyramidal neurons and decreased seizure susceptibility. It thus remains to be elucidated to what extent the plasticity of tonic currents reported here related to the altered contribution of δGABA_A_Rs in PV+ interneurons affects the network excitability and what its impact is on the cognitive and behavioral functions. While δ and α5 subunit-containing GABA_A_Rs are typically present to a larger or smaller extent in different types of neurons, heterosynaptic NMDA-induced plasticity differentially affects these two components of tonic conductance. It is noteworthy that the component of tonic current plasticity related to α5GABA_A_Rs was present in all considered here interneurons (Figures 1–3) as well as in pyramidal 352 neurons (Wyroślak et al., 2021). These observations underscore the importance of the tonic current component mediated by α5GABA_A_Rs which, most interestingly, was plastic in all these cells. However, in the case of PV+ interneurons, NMDA-induced plasticity resulted in an overall decrease in tonic current in spite of increased α5GABA_A_R content, indicating that, as already mentioned, its contribution in these cells was minor.

**Figure 4:**
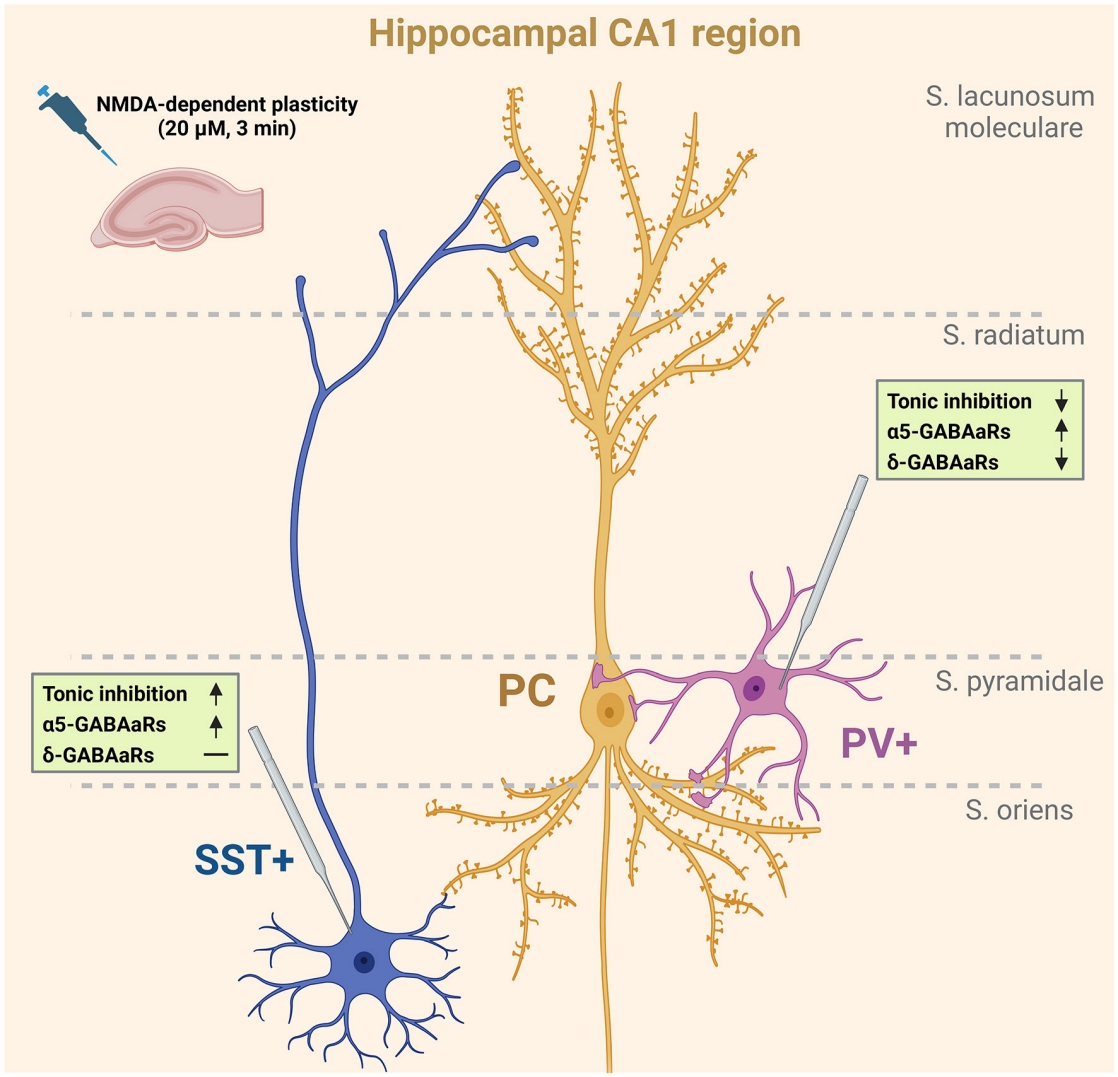
NMDA-dependent plasticity affects tonic currents in PV+ and SST+ interneurons. Schematic representation shows the summary of the results obtained in the present study. Short-term administration of NMDA (3 min, 20 μM) alters tonic currents in the studied types of interneurons. SST+ interneurons innervating pyramidal cells in the stratum lacunosum-moleculare layer are characterized by an α5GABA_A_R-dependent increase in the magnitude of tonic currents upon NMDA treatment. At the same time, the tonic current measured in PV+ interneurons innervating pyramidal cells perisomatically was decreased after plasticity induction. This reduction was dependent on δGABA_A_Rs, but a slight increase in the contribution of α5GABA_A_Rs in tonic current mediation was observed. Created with Biorender.com.

It is worth emphasizing that the role of α5GABA_A_Rs in plastic phenomena is not limited to tonic conductance. A solid body of evidence showed that α5GABA_A_Rs are implicated in GABAergic synapse function, participating in phasic inhibition and thereby controlling postsynaptic excitability (Ali and Thomson, 2008; Zarnowska et al., 2009; Schulz et al., 2018; Magnin et al., 2019; Lodge et al., 2021). Indeed, in a recent study, Davenport et al. (2021) described an interesting form of plasticity in which α5GABA_A_Rs from extrasynaptic zones were relocated into synapses due to the dissociation of these receptors from radixin upon its dephosphorylation. Moreover, Davenport reported that the blockade of α5-GABA_A_Rs in the hippocampus accelerated reversal learning, a test for cognitive flexibility dependent on repeated LTP, providing further evidence that these receptors play a role in cognitive mechanisms. This mechanism has been, however, implicated in the pyramidal neurons, and it remains to be elucidated whether or not it takes place also in interneurons investigated in the present study. In our recent study (Brzdak et al., 2023), we report that a brief treatment with NMDA evoked iLTP in SST+ and iLTD in PV+ interneurons. Thus, the effects on tonic conductance (present study) would thus sum up with analogous changes in phasic signaling in these neurons (Brzdak et al., 2023). As principal cells' spiking and network oscillations were regulated through feedforward and feedback inhibition from PV+ interneurons targeting perisomatic areas, a decrease of tonic inhibition in these interneurons may lead to a more effective inhibition of principal cells (see Figure 4). Thus, the overall synchronization of principal cells activity may be expected to increase (Pouille and Scanziani, 2001). Simultaneously, in the present report, we observed the upregulation of tonic inhibition in somatostatin-containing interneurons that target principal cells' dendrites in stratum lacunosum-moleculare, and this effect is accompanied by increased iLTP of mIPSCs (Brzdak et al., 2023). Considering that SST+ interneurons regulate local dendritic conductances and also excitatory synaptic plasticity, an increase in their tonic inhibition may result in less feedback inhibition onto principal cells (Chiu et al., 2013; Schulz et al., 2018). The opposite directions of synaptic (Brzdak et al., 2023) and primarily extrasynaptic tonic current plasticity of SST+ and PV+ were particularly interesting, considering the crucial impact of these interneurons on the local neuronal network and generation of theta rhythm (Sohal et al., 2009; Royer et al., 2012).

Plastic changes induced by the NMDAR activity may depend on the subtype of these receptors. A recent study, using GluN2A-null rats, has indicated that GluN2A is a major NMDAR subunit in SST+ INs, only partially contributing to NMDA-EPSCs in PV+ cells (Booker et al., 2021). Wu et al. (2021) addressed the involvement of GluN2A- and GluN2B-NMDARs in tonic current regulation in hippocampal neuronal culture and found that GluN2A inhibits and GluN2B promotes α5GABA_A_R internalization, thus providing evidence for the distinct involvement of these NMDA-subunits in regulating tonic inhibitory plasticity. We cannot exclude the possibility that the NMDA-induced increase in tonic current in SST+ INs could involve GluN2A-dependent α5GABA_A_Rs internalization, but this issue would require further studies.

The emerging mechanisms of tonic conductance regulation in different neuronal types by plasticity phenomena related to the components mediated by δ- and α5-GABA_A_Rs appear particularly interesting in the light of growing evidence that, with genetic or pharmacological manipulations, these receptors affect learning and memory formation (Collinson et al., 2002; Saab et al., 2010; Zurek et al., 2012; Whissell et al., 2013b; Cushman et al., 2014; Möhler and Rudolph, 2017). As already mentioned, SST+ and PV+ interneurons are known to play a key role in shaping local circuit excitability as well as in learning and memory and brain pathology (Donato et al., 2013; Caroni, 2015; Ognjanovski et al., 2017; Mikulovic et al., 2018; Tripodi et al., 2018; Donegan et al., 2019; Serrano and Caroni, 2019; Udakis et al., 2020; Asgarihafshejani et al., 2022; Liguz-lecznar et al., 2022), but it remains to be elucidated to what extent the plasticity of tonic conductance at specific interneurons is involved in these functional and cognitive roles.

In conclusion, we present the first evidence that tonic inhibition is a plastic component of GABAergic drive in hippocampal SST+ and PV+ interneurons and have demonstrated that the underlying mechanisms depend on different GABA_A_R subtypes and are cell-type specific.

## Data availability statement

The raw data supporting the conclusions of this article will be made available by the authors, without undue reservation.

## Ethics statement

All experiments were carried out in accordance with the Polish Animal Protection Act (Act of 15 January 2015, changed 17 November 2021; directive 2010/63/EU). The animal study was reviewed and approved by Komisja Bioetyczna przy Instytucie Immunologii i Terapii Doświadczalnej im. Ludwika Hirszfelda Polskiej Akademii Nauk.

## Author contributions

MW conducted experiments, carried out data analysis, and contributed to writing and editing the manuscript. GD provided methodological support and participated in editing the manuscript. JM conceived and supervised the project, procured financial support, participated in designing the experiments, and wrote and edited the final version of the manuscript. All authors contributed to the article and approved the submitted version.
